# Metalorganic Chemical Vapor Deposition Approach to the Synthesis of Perovskite BaCeO_3_ and BaCe_0.8_Y_0.2_O_3_ Thin Films

**DOI:** 10.3390/molecules28083303

**Published:** 2023-04-07

**Authors:** Anna L. Pellegrino, Francesca Lo Presti, Graziella Malandrino

**Affiliations:** Dipartimento di Scienze Chimiche, Università di Catania, and INSTM UdR Catania, Viale A. Doria 6, I-95125 Catania, Italy

**Keywords:** β-diketonate precursors, perovskite, structural characterization, morphology

## Abstract

In the present energetic scenario, the development of materials with high potentiality in the technological fields of energy conversion processes, production and storage of hydrogen, are of great interest in the scientific community. In particular, we report for the first time the fabrication of crystalline and homogeneous barium-cerate-based materials in the form of thin films on various substrates. Starting from the β-diketonate precursor sources Ce(hfa)_3_diglyme, Ba(hfa)_2_tetraglyme and Y(hfa)_3_diglyme (Hhfa = 1,1,1,5,5,5-hexafluoroacetylacetone; diglyme = bis(2-methoxyethyl)ether; tetraglyme = 2,5,8,11,14-pentaoxapentadecane), a metalorganic chemical vapor deposition (MOCVD) approach has been successfully applied to the fabrication of BaCeO_3_ and doped BaCe_0.8_Y_0.2_O_3_ systems in the form of thin films. Structural, morphological and compositional analyses allowed for an accurate determination of the properties of deposited layers. The present approach represents a simple, easily scalable, and industrially appealing process for the production of compact and homogeneous barium cerate thin films.

## 1. Introduction

Perovskite-based structures have attracted huge attention in the last decade mainly due to the discovery of appealing photovoltaic [1] or photocatalytic [2] properties in the halide perovskite systems, either as hybrid or full inorganic phases. On the other hand, perovskite oxides have been studied for decades due to their very interesting properties [3]. In particular, perovskite oxide compounds of alkaline earth elements exhibit unique conducting properties compared to other doped binary oxide materials [4,5,6]. That makes these materials promising for application in various technological fields, such as electrocatalysis [7,8], batteries [9] and ferroelectrics [10]. In particular, BaCeO_3_ and the rare-earth-doped BaCeO_3_ systems have been widely investigated as proton-conducting materials [11]. BaCeO_3_ is an A^2+^B^4+^O_3_-type perovskite, where A occupies a cube-octahedral site and B occupies an octahedral site coordinated by oxygen atoms, to form corner-sharing BO_6_ octahedra. Trivalent doping ions, such as Y, are assumed to occupy the Ce^4+^ sites, introducing oxygen vacancies into the structure and thus causing an enhancement of the protonic conductivity [12,13,14]. The exceptional electrical properties of barium-cerate-based compounds make them attractive candidates for applications as catalysts to produce and store hydrogen [15,16,17,18], as hydrogen sensors [19,20], and as proton conductors in intermediate-temperature solid oxide fuel cells (IT-SOFCs) [21,22,23,24]. Specifically, SOFCs are among the most promising candidates for barium-cerate-based materials. In addition, doping is an efficient strategy to improve the proton-conducting properties of the electrolyte materials [25].

Among the various synthetic approaches for the production of pure BaCeO_3_ phase [26], the most common is the solid-state reaction, which requires high pressure, long reaction time and a high temperature process [15]. Regarding the solution methods, Y. Zhou et al. [27] have recently reported the synthesis of Ru-doped BaCeO_3_ through a sol-gel process, starting from barium nitrate and ceric ammonium nitrate under acidic conditions. However, a calcination process at a higher temperature is required in this case to obtain nanoparticles of pure BaCeO_3_.

Few studies have been reported on the synthesis of barium cerate in the thin film form. Owaku et al. [28] have deposited Y-doped BaCeO_3_ on Al_2_O_3_ (0001) substrates by RF magnetron sputtering. Another physical vapor deposition technique, such as the pulsed laser deposition [29], has also been applied to produce Y-doped BaCeO_3_ films. Spray pyrolysis has been applied to the synthesis of amorphous Gd and Y co-doped barium cerate films, which need further annealing above 700 °C to crystallize [30].

A suited synthetic approach represents a key issue for the massive use of BaCeO_3_-based materials in the form of supported systems, i.e., as thin film or composite arrangement. In this context, the development of an industrially appealing and large-scale process is highly desirable, and thus, metalorganic chemical vapor deposition (MOCVD) represents an appealing method for the production of BaCeO_3_-based materials on large areas with high uniformity of both composition and morphology.

In the present study, we report the fabrication of the BaCeO_3_- and the Y-doped BaCe_0.8_Y_0.2_O_3_ systems through an MOCVD synthetic approach using the β-diketonate adducts of Ce(hfa)_3_diglyme, Ba(hfa)_2_tetraglyme and Y(hfa)_3_diglyme [Hhfa = 1,1,1,5,5,5-hexafluoroacetylacetone; diglyme = bis(2-methoxyethyl)ether and tetraglyme = 2,5,8,11,14-pentaoxapentadecane] as precursor sources. This study represents the first report of the synthesis of BaCeO_3_ and BaCe_0.8_Y_0.2_O_3_ thin films through a full vapor-phase approach.

Preliminarily to the deposition process, thermal analyses have been carried out on the β-diketonate adducts in order to evaluate their applicability in the vapor-phase process and the consistency of their vapor pressures to be used as a single-source precursor. Different operative conditions have been tested in order to obtain pure BaCeO_3_ as compact thin films. The variation of deposition temperature, tested in the 800–950 °C range, has allowed for the selective and reproducible fabrication of pure BaCeO_3_ and BaCe_0.8_Y_0.2_O_3_ thin films. 

## 2. Results and Discussion

An MOCVD approach has been successfully applied to the fabrication of BaCeO_3_ and Y-doped BaCeO_3_ thin films on MgO (100), and yttria-stabilized zirconia (YSZ) (100) substrates, starting from the Ba(hfa)_2_tetraglyme, Ce(hfa)_3_diglyme and Y(hfa)_3_diglyme complexes. The suitability as MOCVD precursors of the above-mentioned adducts, previously synthesized in our group [31,32,33], has been assessed through their application to the fabrication of various materials: (i) the Ce(hfa)_3_diglyme has been applied to the growth of binary CeO_2_ thin films [34]; (ii) the Ba(hfa)_2_tetraglyme has been used to fabricate the piezoelectric Ba and Ti co-doped BiFeO_3_ textured films [35]; and (iii) the Y(hfa)_3_diglyme has been applied for the fabrication of the YAlO_3_ films [36].

MgO and YSZ substrates have been chosen because YSZ is the most commonly used ceramic component in the anode cermet in SOFCs. Nevertheless, the quantification of Y through EDX would have been hampered by the presence of Y in the substrate. This is the reason why MgO has also been used to allow for the EDX analysis and the quantification of all the elements present in the film. 

### 2.1. Thermal Characterization of the Precursors

In the present work, the Ba(hfa)_2_tetraglyme, Ce(hfa)_3_diglyme and Y(hfa)_3_diglyme complexes have been applied for the first time as metalorganic precursors for the MOCVD deposition of pure and Y-doped BaCeO_3_ phase films.

**Figure 1:**
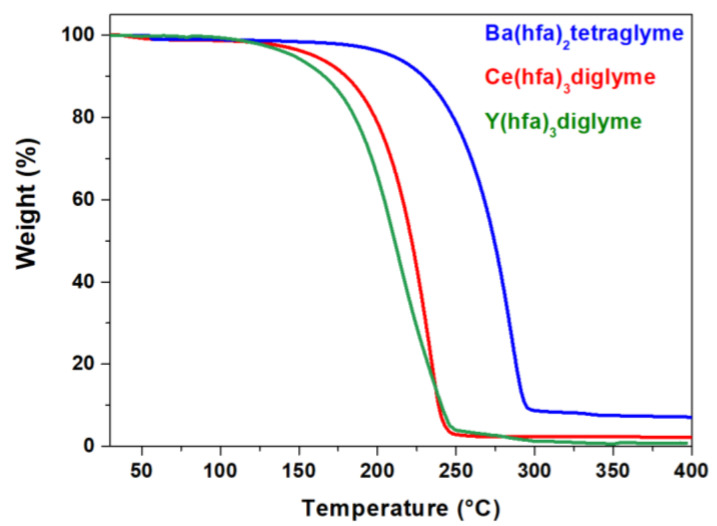
TGA profiles of Ba(hfa)_2_tetraglyme, Ce(hfa)_3_diglyme and Y(hfa)_3_diglyme under N_2_ flow at atmospheric pressure, in the temperature range 25–400 °C.

The thermal behavior of the starting precursors has been deeply analyzed in order to assess the best MOCVD setup and operational parameters for the synthesis of pure and Y-doped BaCeO_3_ thin films. The thermal analyses of the as-synthesized precursors have been investigated using thermogravimetric (TG) measurements, and the relative curves have been reported in Figure 1. In particular, the TG curves of the Ba, Ce and Y adducts show high stability of up to 150 °C, with a single-step mass loss associated with the adduct vaporizations at 220 °C, 232 °C and 265 °C for Y(hfa)_3_diglyme, Ce(hfa)_3_diglyme and Ba(hfa)_2_tetraglyme, respectively. The residues after the vaporization process are around 2% in weight for the Ce and Y adducts, while it is about 10 % for the Ba one at 350 °C. This evidence suggests an excellent thermal behavior of all the metalorganic adducts with a clean vaporization process during the heating treatment, pointing to a suitable thermal characteristic for conventional MOCVD processes. Based on the TG curves, it is evident that the Ba adduct is less volatile than the Ce and Y complexes. Thus, according to present data, the vaporization temperatures at reduced pressure have been fixed at 145 °C for the Ba(hfa)_2_tetraglyme crucible, and at 130 °C for the pure Ce(hfa)_3_diglyme or the mixture of Ce(hfa)_3_diglyme and Y(hfa)_3_diglyme adducts during the deposition processes. Notably, the possibility of using the Ce and Y adducts as a mixture is conceivable due to their similar thermal behaviors, which point to a single-source performance. In fact, in a previous work [37], it has been demonstrated, for analogous precursor mixtures, that the presence of the same ligands in the adducts allows for hypothesizing single-source behavior and excluding a potential ligand exchange issue.

### 2.2. Deposition of BaCeO_3_ Films

An in-depth study has been conducted on the deposition process in order to obtain the BaCeO_3_ pure phase in the form of homogeneous and compact thin films. In particular, the effects of the deposition temperature and the substrate nature have been investigated.

**Figure 2:**
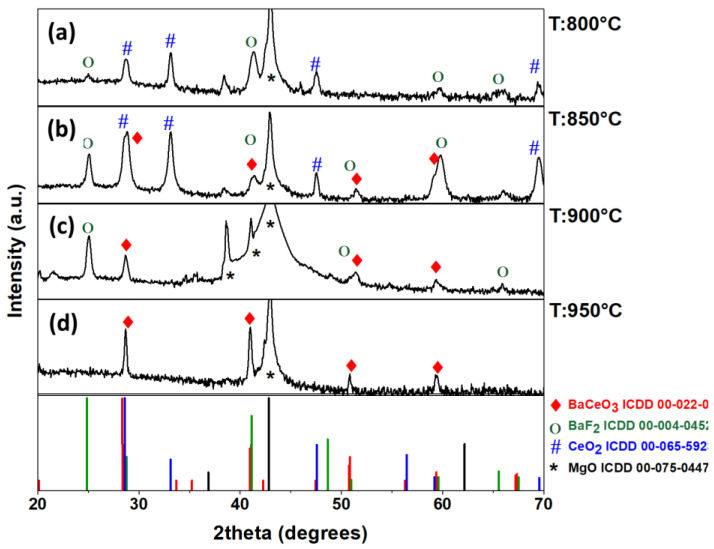
XRD patterns of Ba–Ce-based thin films grown on the MgO (100) substrate at different deposition temperatures: (**a**) 800 °C; (**b**) 850 °C; (**c**) 900 °C; and (**d**) 950 °C.

Structural characterization through XRD analyses displays the formation of different crystalline phases, depending on the deposition temperature. Figure 2 reports the patterns recorded for different samples deposited on MgO (100) substrates as a function of the deposition temperatures. At a lower temperature, i.e., 800 °C and 850 °C (Figure 2a,b), a mixture of different phases has been detected. In particular, peaks associated with the CeO_2_ and BaF_2_ have been found under both deposition temperatures. Furthermore, the presence of certain peaks (shown in red in the Figure 2b) suggests the formation of a slight amount of the BaCeO_3_ phase. However, the detection of this phase cannot be attributed univocally. At a higher temperature of 900 °C (Figure 2c) instead, the formation of the BaCeO_3_ phase has been confirmed by the peaks at 28.70°, 50.92° and 59.24°, related to the 002, 213/231 and 004/422 reflections, respectively. Under these conditions, however, impurities of BaF_2_ have been found as well. The peaks at 38.64° and 41.06° are related to 200 reflection of the MgO (100) substrate arisen from the diffraction of the Cu K_β_ and W L_α1_ line, respectively. Lastly, the film obtained at 950 °C presents only peaks associated with the formation of pure and polycrystalline BaCeO_3_ perovskite phase at 28.66°, 40.98°, 50.86° and 59.40°, which correspond to the 002, 022/400, 213/231 and 004/422 reflections (Figure 2d) (International Centre Diffraction Data (ICDD) no. 00-022-0074). The presence of peaks due to differently oriented grains indicate the formation of polycrystalline films with a random grain growth.

The crystallite dimension, derived through the Scherrer equation, is 42 nm for the BaCeO_3_ films.

The observed BaCeO_3_ pattern has been attributed to the orthorhombic structure, with parameters *a* = 8.7790 Å, *b* = 6.2140 Å and *c* = 6.2360 Å. The orthorhombic structure has been reported to be more thermodynamically stable at room temperature [38]. This observation finds counterpart in the classification proposed by Goldschmidt [39]. In fact, the BaCeO_3_ has a “tolerance factor” (*t*) value of 0.85, as calculated through Equation (1):

(1) $$t=\frac{\left(R_{A}+R_{O}\right)}{\sqrt{2}\left(R_{B}+R_{O}\right)}$$

where *R_A_*, *R_B_*, and *R_O_* are the ionic radii of Ba^2+^, Ce^4+^ and O^2−^ in the BaCeO_3_ structure. It is worthy to note that to calculate the *t* factor, the crystal ionic radii of six coordinated ions have been applied [40]. In fact, Goldschmidt applied the tolerance factor formula using his own ionic radii [39], which can be assimilated to six coordinated ionic radii reported in the Shannon table [40]. Thus, to correctly evaluate the tolerance factor, six coordinated ionic radii values have to be considered [41] independently of the real coordination of the ion in the perovskite structure. The calculated tolerance factor value of 0.85 is indicative of a distorted structure and fits perfectly with the orthorhombic phase, stable at room temperature, while an ideal cubic structure is usually defined by a tolerance factor ranging from 0.9 to 1 [39].

The trend observed through XRD analysis points to the formation of binary phases of CeO_2_ and BaF_2_ rather than the BaCeO_3_ perovskite at lower deposition temperatures. This result can be rationalized considering that the oxide and fluoride phases are usually stabilized at a lower temperature, while the perovskite requires more energy, and thus a higher temperature to form. 

**Figure 3:**
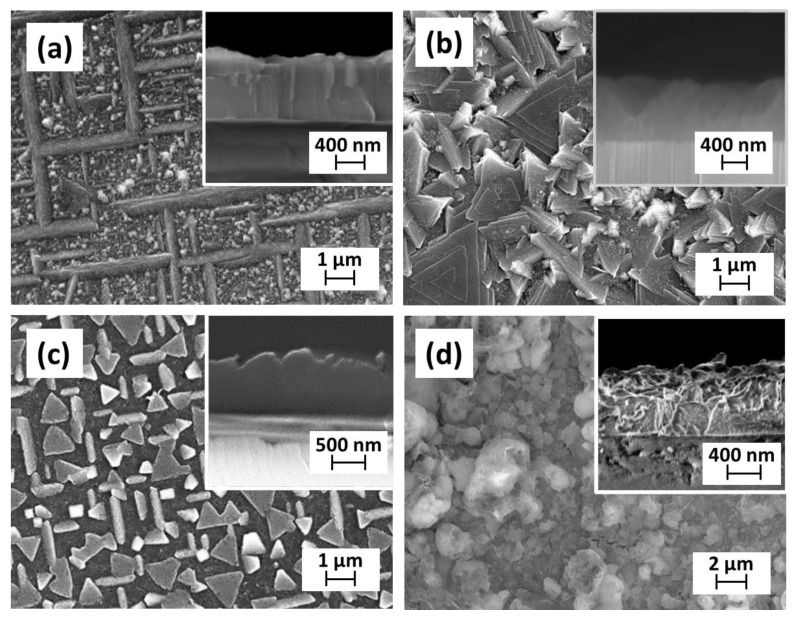
FE-SEM images of Ba–Ce-based thin films grown on the MgO (100) substrate at different deposition temperatures: (**a**) 800 °C; (**b**) 850 °C; (**c**) 900 °C; and (**d**) 950 °C. Insets in the FE-SEM images report the cross-section of the corresponding films.

As a consequence, the differences in phase composition are also reflected in the morphology of the layers, as observed through the FE-SEM characterization. In Figure 3a, the film obtained at 800 °C shows the formation of a nanostructured layer, with grains of hundreds of nanometres uniformly distributed between ordered rod/plate-like grains of a few microns. The film thickness of about 875 ± 25 nm is visible in the inset of Figure 3a. The sample obtained at 850 °C, reported in Figure 3b, presents a surface characterized by irregular grains, in which several triangular-shaped grains of the order of microns are observed. In particular, these triangular spiral nanoplates are indicative of a spiral-like growth [42], probably associated with the CeO_2_ growth and in accordance with the intense CeO_2_ 111 reflection observed at 28.85° in the related pattern. This quite peculiar morphology is also visible in the cross section (inset of Figure 3b), where a thickness ranging from 700 to 930 nm can be considered. Smaller and uniformly distributed triangular grains are instead displayed in Figure 3c for the sample obtained at 900 °C. 

It is interesting to observe that the films, arising from an island growth, are formed by nanostructures and the compact layer covering the substrate underneath the nanostructures. The cross-sectional image, reported in the inset in Figure 3c, indicates a thickness of about 880 ± 40 nm. Finally, in Figure 3d, at the highest temperature of 950 °C, the surface is formed of quite spheroidal grains of about 1 µm. The related FE-SEM cross-sectional image of the pure BaCeO_3_ film shows the formation of a compact layer with coalesced grains (inset in Figure 3d). A thickness of about 810 ± 30 nm has been found, thus a growth rate of 13.5 nm/min has been determined. This result suggests that the growth of the BaCeO_3_ needs to be optimized in terms of deposition temperature and a high temperature of 950 °C is needed in this process to stabilize the pure phase. Actually, the conventional CVD approach for the synthesis of perovskite-based materials usually requires such a range of temperatures [41].

**Figure 4:**
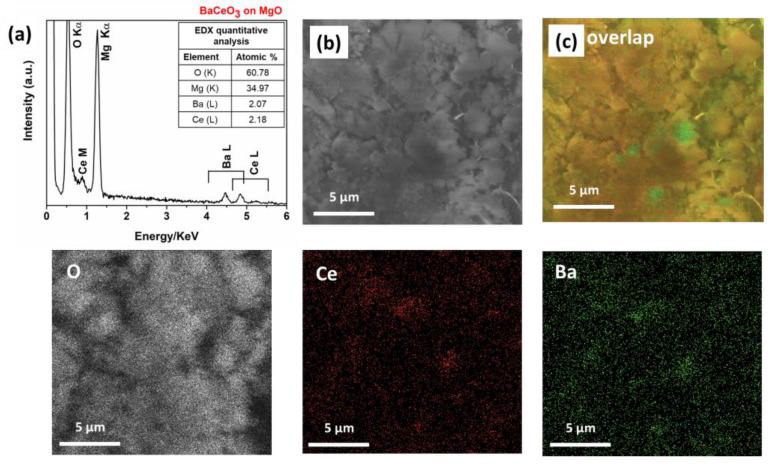
(**a**) EDX spectrum, (**b**) relative FE-SEM image, EDX elementary maps of O, Ce and Ba elements, and (**c**) overlapped map image of the BaCeO_3_ film deposited on MgO at 950 °C.

In order to address the composition and the purity of the BaCeO_3_ thin film, an EDX analysis has been executed and reported in Figure 4a, where the spectrum shows the characteristic peaks of barium, cerium and oxygen elements. In particular, the Ba shows peaks in the range of 4.4–5.5 keV due to the L-lines, Ce shows peaks at 0.88 keV (M_α_) and in the range of 4.7–5.9 keV due to the L-lines, while O shows a peak at 0.53 keV due to the K_α_. The EDX quantitative analysis confirms the correct stoichiometry of the pure BaCeO_3_ films, with a ratio of Ba to Ce of about 1:1 on the whole surface. The signal of magnesium is due to the substrate. The absence of the peaks at 0.277 keV and 0.677 keV excludes the presence of C and F within the detection limit of the technique of about 1%, thus pointing to a clean decomposition of the precursors under these deposition conditions.

Moreover, the homogeneity of the film has been assessed through EDX maps on the pure BaCeO_3_ films deposited on the MgO substrate (Figure 4b,c). For this aim, a large area has been analyzed in order to confirm the homogeneity of the systems. In Figure 4b, the colored maps of Ba, Ce and O elements have been reported. The maps indicate a uniform distribution of the elements in the BaCeO_3_ layer, as confirmed in the overlapped image (Figure 4c).

**Figure 5:**
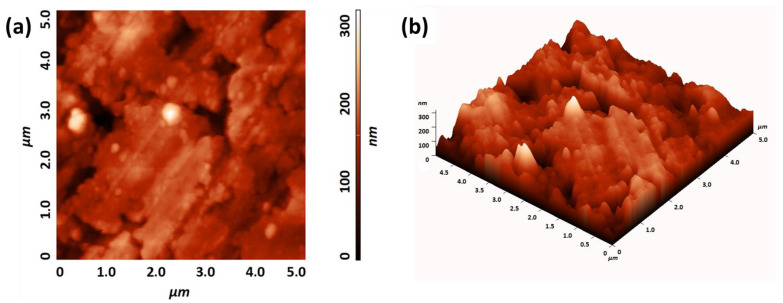
(**a**) Two-dimensional and (**b**) 3D AFM topographical images (RMS roughness of 29.4 nm on 2.5 × 2.5 µm area) of the BaCeO_3_ film grown on the MgO substrate at a deposition temperature of 950 °C.

Finally, the atomic force microscopy (AFM) characterization of the BaCeO_3_ film grown on MgO (Figure 5a,b) confirms the homogeneity of the layer, with coalesced grains on a large area of 5 μm × 5 µm, and a root mean square (RMS) roughness of about 29.4 nm (measured on an area of 2.5 μm × 2.5 μm) being evaluated.

### 2.3. MOCVD Deposition of Y-Doped BaCeO_3_ Films

In the second part of this work, the optimized procedure has been tested for the growth of Y-doped BaCeO_3_ on the YSZ (100) substrate, having an *a*-axis parameter of 5.1390 Å. The deposition temperature was fixed at 950 °C and the Y doping percentage at 20%. The Y doping value has been chosen based on both theoretical and experimental studies that report better conducting properties for a percentage around 20% of rare-earth ion doping. For example, in the first-principles calculations [13,14], the studied compositions were BaCe_0.8_Y_0.1_Ni_0.04_Sm_0.06_O_3-δ_ and BaCe_0.75_Y_0.125_Nd_0.125_O_3-δ_, respectively, while experimental studies indicate a rare-earth doping percentage ranging from 10% (BaCe_0.9_Y_0.1_O_3_, [24,28,29]) to 30% composition (BaCe_0.7_Gd_0.1_Y_0.2_O_2.9_, [30]).

**Figure 6:**
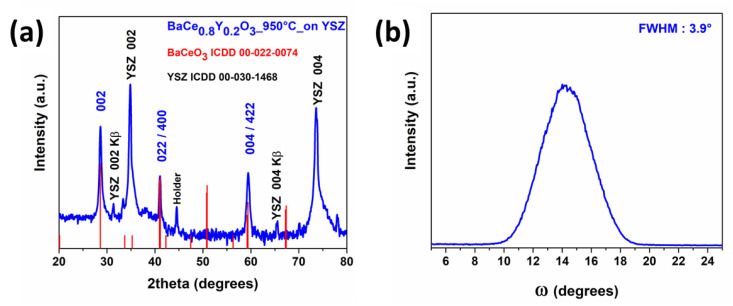
(**a**) XRD pattern and (**b**) rocking curve of the 002 reflection of Y-doped BaCeO_3_ thin film grown on the YSZ (100) substrate at a deposition temperature of 950 °C.

The results of the synthesized films are reported in Figure 6. The XRD pattern (Figure 6a) shows the formation of the Y-doped BaCeO_3_ phase due to the presence of peaks at 28.76°, 41.20° and 59.48°, associated with the 002, 022/400 and 004/422 reflections, respectively. In addition, the signals at 31.3° and 65.7° come from the K_β_ of the YSZ-002 and the K_β_ of the YSZ-004, respectively. The peak at 44.6° rises from the holder of the XRD machine. The pattern has been analysed using the peaks of the YSZ substrate as the internal standard to correctly align the peak positions. This procedure allowed us to confirm that the peaks of the Y-doped BaCeO_3_ film correspond perfectly to the peak positions of the undoped phase (ICDD no. 00-022-0074). This observation is in accordance with the similar ionic radius of the Y^3+^ ion of 0.90 Å (six-coordination), compared to the Ce^4+^ ionic radius value of 0.87 Å (six-coordination), all of them taken from the Shannon table for a six-fold coordination [40].

The use of the YSZ substrate induces a certain orientation growth along the <001>, as confirmed by the observation of the rocking curve of the 002 peak. On the other hand, the large mismatch between the film and substrate lattice parameters is responsible for the presence of additional peaks, such as the 022/400. 

Furthermore, considering that the sample is mainly <00l>-oriented, in order to study the out-of-plane alignment of the deposited film, the rocking curve of the 002 reflection at 2θ = 28.76° has been recorded (Figure 6b). The rocking curve full-width half-maximum (FWHM) value of 3.9° confirms a certain out-of-plane alignment of the Y-doped BaCeO_3_ film, mainly grown along the <001> direction on YSZ (100) substrate. 

Crystallite dimension determined through the Scherrer equation is 31 nm for the Y-doped BaCeO_3_ films. This value may be slightly underestimated, since in the Scherrer’s formula peak broadening due to strain is not taken into account. This value may be compared to that of the pure BaCeO_3_ phase of 42 nm. These findings may be related to the different substrate nature and parallel what is observed in the FE-SEM images, i.e., larger grains are found for the pure film deposited on MgO. It is worth noting that the FE-SEM grain dimensions are much larger than the crystallite dimensions, since the former may originate from the coalescence of various crystallites.

**Figure 7:**
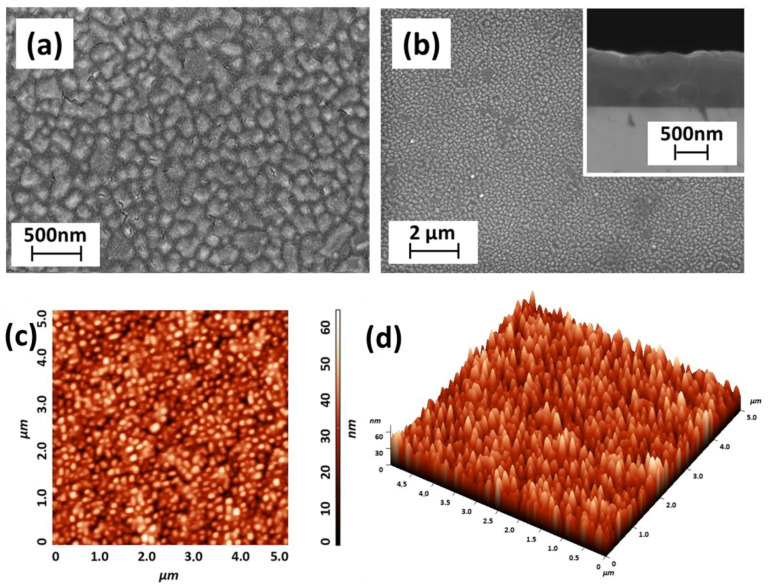
(**a**,**b**) FE-SEM images and (**c**) 2D and (**d**) 3D AFM topographical image (RMS roughness of 10.2 nm on 2.5 × 2.5 µm area) of BaCe_0.8_Y_0.2_O_3_ thin film grown on the YSZ (100) substrate at a deposition temperature of 950 °C. Inset in (**a**) reports the cross-sectional image of the film.

The morphology reported in Figure 7a,b displays the formation of a uniform layer on a large area, with quite evident nanostructured features and rectangular grains of the order of 50–100 nm. In contrast to the analogous undoped film deposited on MgO (100) (see Figure 3d), the YSZ (100) substrate drives the film growth with well-defined and regular grains.

The inset in Figure 7a shows a cross-sectional image of the sample, with a thickness of 920 ± 15 nm and a growth rate of about 15 nm/min, similar to the one found for the undoped system.

The AFM image of the BaCeO_3_ sample grown on YSZ (100) (Figure 7c,d) shows a very uniform surface with very small rounded grains, confirming the homogeneity of the layer on the large area of 5 μm × 5 µm. The root mean square (RMS) roughness measured on an area of 2.5 μm × 2.5 μm is 10.2 nm, smaller than that observed for the sample deposited on MgO. This observation has a counterpart in the different morphologies observed in the two cases.

**Figure 8:**
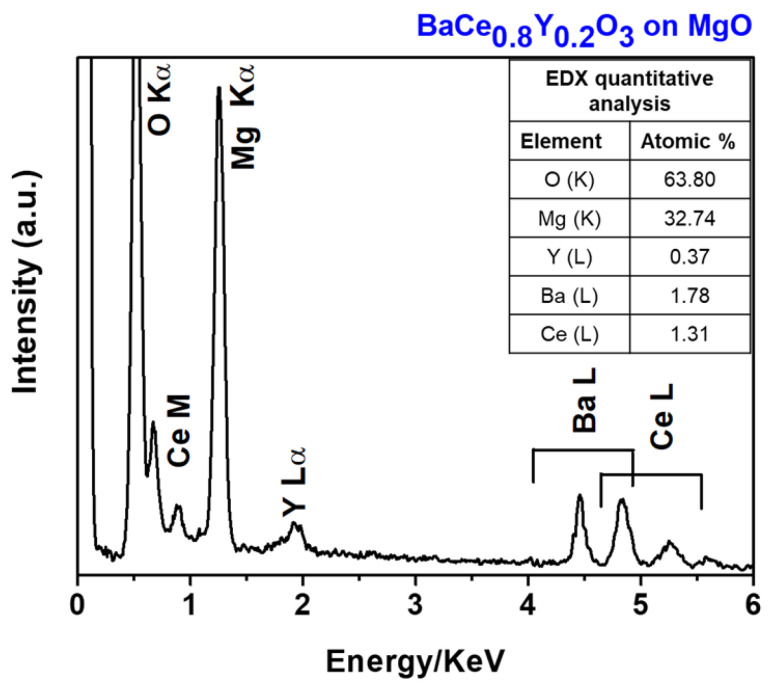
EDX spectrum of the BaCe_0.8_Y_0.2_O_3_ film grown on the MgO substrate at a deposition temperature of 950 °C.

Additionally, in order to address the compositional characterization of the deposited films, quantitative measurements have been conducted through EDX analyses of BaCe_0.8_Y_0.2_O_3_ film grown on the MgO substrate. The EDX spectrum in Figure 8 confirms that the atomic ratio of Ba, Ce and Y of the sample is practically identical to the nominal composition of the starting precursor sources. In fact, the stoichiometry of the film has been found to be 1:0.78:0.22 for the Ba: Ce: Y elements. Additionally, the homogeneity of the film over large areas has been assessed through the EDX maps.

**Figure 9:**
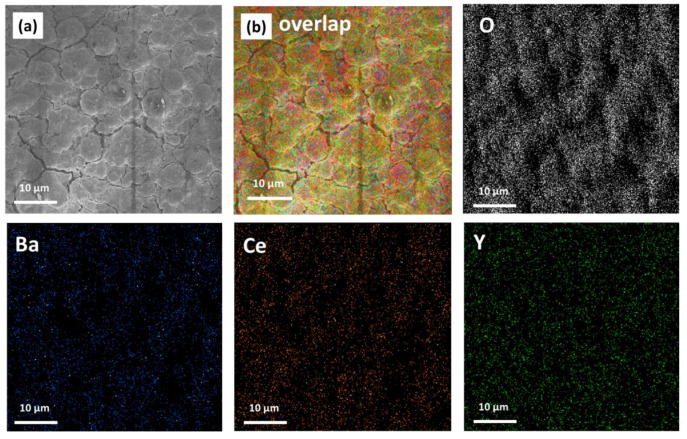
(**a**) FE-SEM image, the EDX elementary maps of O, Ba, Ce and Y elements, and (**b**) the overlapped map image of the BaCe_0.8_Y_0.2_O_3_ film grown on the MgO substrate at a deposition temperature of 950 °C.

In Figure 9, the relative maps present an even distribution of the Ba, Ce and O elements, and the Y dopant is also homogenously distributed all over the Y-doped BaCeO_3_ thin film (Figure 9b). This evidence is an important aspect to assess the suitability of the reported process for the formation of doped BaCeO_3_ systems over large areas. In fact, the uniform distribution of the doping ion is of paramount importance for the final functional properties of the material.

Structural and morphological properties of present films may be compared to films deposited through spray coating. With respect to the spray-deposited samples presently, MOCVD-deposited films are more crystalline at about the same temperature, and have a very compact morphology, as also visible in the cross-sectional FE-SEM images.

## 3. Materials and Methods

### 3.1. Reagents and Precursor Syntheses

Commercial barium hydroxide [Ba(OH)_2_•8H_2_O], cerium nitrate [Ce(NO_3_)_3_•6H_2_O] or yttrium acetate hydrate [Y(CH_3_COO)_3_•xH_2_O], and Hhfa (Hhfa = 1,1,1,5,5,5-hexafluoroacetylacetone) were purchased from STREM Chemicals Inc. Bischheim (France), while diglyme and tetraglyme (diglyme = bis(2-methoxyethyl)ether and tetraglyme = 2,5,8,11,14-pentaoxapentadecane) were purchased from Sigma-Aldrich (Merck KGaA, Germany). All chemicals were used without any further purification.

The Ba(hfa)_2_tetraglyme, Ce(hfa)_3_diglyme and Y(hfa)_3_diglyme compounds were synthesized by reacting the barium hydroxide, cerium nitrate hydrate, or yttrium acetate hydrate, respectively, with the ligands Hhfa and polyether, such as tetraglyme (for Ba) and diglyme (for Ce and Y). 

#### 3.1.1. Ba(hfa)_2_tetraglyme

The synthesis was carried out following the same procedures reported in [31], with the only difference being using dichloromethane instead of toluene. [Ba(OH)_2_•8H_2_O] was first suspended in dichloromethane, and the tetraglyme was added to the suspension. H-hfa was added after 10 min and the mixture was refluxed under stirring for 1 h. After solvent evaporation, the complex appears in the form of non-hygroscopic white crystals.

#### 3.1.2. Ce(hfa)_3_diglyme

The compound was synthesized from the cerium nitrate [Ce(NO_3_)_3_•6H_2_O], diglyme and Hhfa, following the procedure reported in [32]. Yellow crystals were recovered after evaporation of the solvent, and they were then washed with pentane.

#### 3.1.3. Y(hfa)_3_diglyme

The compound was synthesized following a modified procedure of that reported in [33], starting from the yttrium acetate hydrate [Y(CH_3_COO)_3_•xH_2_O] suspended in dichloromethane. Diglyme was added to the suspension and H-hfa was added under vigorous stirring after 10 min. The mixture was refluxed under stirring for 1 h. Non-hygroscopic transparent crystals were recovered after solvent evaporation, and they were then washed with pentane. 

### 3.2. MOCVD Depositions

Depositions were performed in a horizontal, hot-wall reactor under reduced pressure. Argon flow (150 sccm) and water-vapor-saturated oxygen flow (800 sccm) were used as the carrier gas and reacting gas, respectively. The flows were introduced in proximity to the reaction zone and were controlled using the MKS 1160 flow controller units (MKS Instruments, Andover, MA, USA). The vacuum inside the reactor was maintained through a scroll pump unit and monitored at the value of 4 Torr using MKS Baratron 122AAX (MKS Instruments, Andover, MA, USA). The films were deposited on MgO (100) and YSZ (100) at the deposition temperature ranges of 800–950 °C. The precursor sources were kept at temperatures of 130 °C and 145 °C for the Ce(hfa)_3_diglyme and Ba(hfa)_2_tetraglyme, respectively, for an efficient vaporization process. For the deposition of Y-doped BaCeO_3_ thin films, Ce(hfa)_3_diglyme and Y(hfa)_3_diglyme precursors were mixed in a stochiometric ratio of 0.8:0.2 and kept at 130 °C, while the Ba precursor was maintained at 145 °C. The deposition time was fixed at 1h.

### 3.3. Characterization

Thermogravimetric analyses of the precursors were executed using the Mettler Toledo TGA2 equipment (Mettler-Toledo S.p.A., Milano, Italy) and STAR^e^ software. The dynamic thermal studies were performed under purified nitrogen flow (50 sccm) at atmospheric pressure, with a 5 °C min^−1^ heating rate. The weights of the samples were between 7 and 10 mg.

Structural characterization was performed using a Smartlab diffractometer (Rigaku, Tokyo, Japan), in the Bragg–Brentano mode operating at 45 kV and 200 mA, equipped with a rotating anode of Cu K_α_ radiation. The patterns were recorded in the range of 20–70°, using a step of 0.02°. Film morphologies were investigated through field emission scanning electron microscopy (FE-SEM) ZEISS SUPRA 55 VP (ZEISS, Jena, Germany). The films were Au-coated before the FE-SEM characterizations. All the FE-SEM images were recorded using a beam energy of 15 keV and a working distance of about 3.5 mm, using the in-lens secondary electron detector. Several regions were investigated in order to assess the uniformity of the film morphologies. The atomic composition of the samples was determined by performing energy dispersive X-ray (EDX) analysis, using an INCA-Oxford windowless detector (Oxford Instruments, Abingdon, UK), with a resolution of 127 eV as the FWHM of Mn Kα. For each sample, three different regions were analysed to confirm the compositional homogeneity. The AFM images were obtained in the contact mode. Before and after each measurement the noise level was 0.01 nm. Several regions were analyzed in order to obtain the most representative region of each sample and an accurate value of the root mean square (RMS) roughness.

## 4. Conclusions

In summary, we present for the first time a metalorganic chemical vapor deposition approach to the synthesis of pure and Y-doped BaCeO_3_ perovskite crystalline phase in the form of thin films. The focus of the work has been devoted to the fine tailoring of the operative conditions, which allow for selectively and reproducibly obtaining the BaCeO_3_ perovskite structure as a pure phase. Indeed, a comprehensive study of the synthetic approach has been described, paying attention to the effects of the deposition temperature on the composition of the final films. In particular, the deposition temperature fixed at 950 °C and the choice of an appropriate substrate are key parameters in the fabrication process. The Ce(hfa)_3_diglyme and Ba(hfa)_2_tetraglyme complexes have been successfully applied as precursor sources for the Ce and Ba components, respectively. Furthermore, the BaCe_0.8_Y_0.2_O_3_ phase has been successfully obtained using the optimized procedure and a mixture of the Ce and Y precursors, with Ba used as a separate source. The advantages of the present approach are mainly related to the tunability of the process in terms of both doping nature and concentration, phase and morphology features for the production of pure, uniform and compact BaCeO_3_ films on the oriented substrates.

This research represents a preliminary and very important step for the production of barium cerate films and opens the way for an upgrading of the MOCVD process for the production of perovskite functional materials through a fine tuning of processing parameters. In addition, the present MOCVD process can be applied on a large scale, and thus paves the way for a consistent production of Y-doped BaCeO_3_ thin films, the material of interest for IT-micro SOFCs technologies.

## Data Availability

Data supporting results are available from the authors.

## Sample Availability

Samples of the compounds are available from the authors.
